# Physical and Functional Characterization of PLGA Nanoparticles Containing the Antimicrobial Peptide SAAP-148

**DOI:** 10.3390/ijms24032867

**Published:** 2023-02-02

**Authors:** Muhanad Ali, Miriam E. van Gent, Amy M. de Waal, Bjorn R. van Doodewaerd, Erik Bos, Roman I. Koning, Robert A. Cordfunke, Jan Wouter Drijfhout, Peter H. Nibbering

**Affiliations:** 1 Department of Infectious Diseases, Leiden University Medical Center, 2300 RC Leiden, The Netherlands; 2 Department of Cell and Chemical Biology, Leiden University Medical Center, 2300 RC Leiden, The Netherlands; 3 Department of Immunology, Leiden University Medical Center, 2300 RC Leiden, The Netherlands

**Keywords:** antimicrobial peptide, skin infection, PLGA, nanoparticle, drug delivery system, bacterial, biofilm

## Abstract

Synthetic antimicrobial and antibiofilm peptide (SAAP-148) commits significant antimicrobial activities against antimicrobial resistant (AMR) planktonic bacteria and biofilms. However, SAAP-148 is limited by its low selectivity index, i.e., ratio between cytotoxicity and antimicrobial activity, as well as its bioavailability at infection sites. We hypothesized that formulation of SAAP-148 in PLGA nanoparticles (SAAP-148 NPs) improves the selectivity index due to the sustained local release of the peptide. The aim of this study was to investigate the physical and functional characteristics of SAAP-148 NPs and to compare the selectivity index of the formulated peptide with that of the peptide in solution. SAAP-148 NPs displayed favorable physiochemical properties [size = 94.1 ± 23 nm, polydispersity index (PDI) = 0.08 ± 0.1, surface charge = 1.65 ± 0.1 mV, and encapsulation efficiency (EE) = 86.7 ± 0.3%] and sustained release of peptide for up to 21 days in PBS at 37 °C. The antibacterial and cytotoxicity studies showed that the selectivity index for SAAP-148 NPs was drastically increased, by 10-fold, regarding AMR *Staphylococcus aureus* and 20-fold regarding AMR *Acinetobacter baumannii* after 4 h. Interestingly, the antibiofilm activity of SAAP-148 NPs against AMR *S. aureus* and *A. baumannii* gradually increased overtime, suggesting a dose–effect relationship based on the peptide’s in vitro release profile. Using 3D human skin equivalents (HSEs), dual drug SAAP-148 NPs and the novel antibiotic halicin NPs provided a stronger antibacterial response against planktonic and cell-associated bacteria than SAAP-148 NPs but not halicin NPs after 24 h. Confocal laser scanning microscopy revealed the presence of SAAP-148 NPs on the top layers of the skin models in close proximity to AMR *S. aureus* at 24 h. Overall, SAAP-148 NPs present a promising yet challenging approach for further development as treatment against bacterial infections.

## 1. Introduction

Antimicrobial resistance (AMR) is a leading cause of death worldwide. The World Health Organization (WHO) has declared AMR as one of the top 10 global health threats facing humanity. In 2019, bacterial AMR was responsible for 1.27 million deaths worldwide, particularly in low-income countries [1,2], and the development of effective antimicrobial agents is a top priority in the fight against AMR bacterial infections. Hence, a library of synthetic novel antimicrobial peptide (AMP) analogues was designed, inspired by the sequence of endogenous human cathelicidin LL-37 and screened for antibacterial and anti-biofilm properties against AMR bacteria. The lead peptide, SAAP-148, underwent further testing against AMR bacteria belonging to the ESKAPE panel (*Enterococcus faecium*, *Staphylococcus aureus*, *Klebsiella pneumoniae*, *Acinetobacter baumannii*, *Pseudomonas aeruginosa*, and *Enterobacter* species)—planktonic, in biofilms, or persisters—and tested in biologically relevant media (i.e., plasma or urine) [3]. SAAP-148 was found to be highly effective in vitro against AMR bacteria and did not display resistance, inhibited biofilm formation, and eradicated established biofilms and persisters. More interestingly, SAAP-148 treatment eliminated acute and established *S. aureus* and *A. baumannii* biofilm infections in an ex vivo human burn wound model and an in vivo superficial murine tape-stripped model [3,4]. Surprisingly, SAAP-148 displayed poor antimicrobial activity against AMR *S. aureus* in an in vivo partial thickness wound model in rats [5]. In addition, SAAP-148 expressed cytotoxicity, in vitro and ex vivo, to mammalian cells at therapeutic concentrations, while the peptide’s stability was reduced in the wound environment [4,6]. Hence, application of SAAP-148 in more complex wound models will require the development of a customized drug delivery system (DDS) to achieve improved peptide stability and prolonged bioactivity in more complex wound models.

Poly(lactic-co-glycolic) acid (PLGA) is a biodegradable and biocompatible synthetic polymer with the ability to encapsulate therapeutic agents in spherical nanoparticles (NPs). PLGA NPs provide several advantageous properties for the delivery of therapeutic agents, with the most important being the induction of controlled release over several days, protection against pre-mature degradation by hydrolytic enzymes and proteases, reduction in the cytotoxicity to mammalian cells, the possibility for surface functionalization to target the site of infection, or co-delivery with a secondary therapeutic biomolecule (e.g., antibiofilm, antibiotics, or anti-inflammatory) [7]. PLGA degrades to alpha-hydroxy-acids (AHAs), namely lactic acid and glycolic acid, which activate several skin rejuvenating functions by epidermal remodeling and desquamation, which stimulate wound healing [8,9]. Of note, AHAs are a major part of cosmetics because they promote the production of hyaluronic acid, collagen, and elastin to reverse the signs of ageing on the skin [10]. In addition, AHAs were shown to promote wound healing by inducing cell proliferation and differentiation [11,12]. Chereddy et al. showed accelerated wound healing of LL-37-PLGA NPs in full thickness excision wounds compared to LL-37 or PLGA alone [13]. PLGA NPs are commonly formulated using the solvent evaporation double emulsion technique to encapsulate hydrophilic/amphiphilic peptides in PLGA NPs [14]. This technique offers several advantageous properties, including easily adjustable formulation parameters to manufacture NPs with desirable physiochemical properties (e.g., size, charge, dispersity, and loading).

In this study, we aimed to physically and functionally characterize SAAP-148 formulated in PLGA NPs (SAAP-148 NPs) by studying their colloidal physiochemical properties and antimicrobial activity against AMR bacteria and such bacteria in biofilms. In addition, the antibacterial activity of the SAAP-148 NPs and colocalization with bacteria were investigated in 3D human skin equivalents (HSEs). The selectivity index was used to quantify the therapeutic activity of SAAP-148 NPs, compared to free peptides, by calculating the ratio between the cytotoxic concentration against skin cells and lethal antibacterial concentration in biologically relevant conditions. Determination of the selectivity index provides an estimation of the relative safety of a drug product for application in the clinic.

## 2. Results

### 2.1. Physiochemical Characterization of SAAP-148 NPs

**Figure 1:**
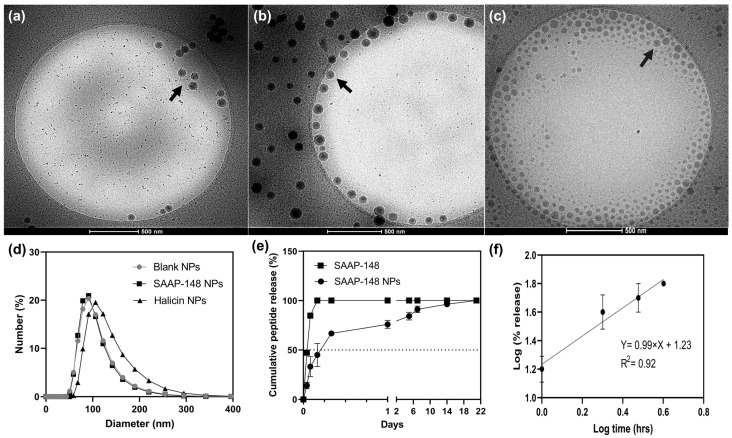
Physiochemical properties of PLGA NPs formulated using the solvent evaporation double emulsion technique. Representative images acquired with Cryo-EM of (**a**) blank PLGA NPs, (**b**) SAAP-148 NPs, and (**c**) halicin NPs. Scale bar = 500 nm. Arrows indicate spherical nanoparticles formulated with the double emulsion technique under identical formulation parameters. (**d**) The particle size distribution obtained with dynamic light scattering (DLS) representing (circles) blank PLGA NPs, (squares) SAAP-148 NPs, and (triangles) halicin NPs. (**e**) Peptide release profile over a period of 21 days, from (circles) SAAP-148 NPs and SAAP-148 solution (squares), using a Float-A-Lyzer in PBS at 37 °C. The concentration of peptide in the outer compartment of the Float-A-Lyzer, filled with free peptide in the inner compartment and expressed as % of the initial concentration, is indicated by squares. The dashed line indicates the 50% peptide release cut-off point. (**f**) Fitting the first log 60% of peptide release (%) by log time (h) into the Korsmeyer–Peppas Model to determine mechanism governing release from SAAP-148 NPs. Data are provided as the mean and standard deviation of three independent experiments performed in duplicates.

Cryo electron microscopy (cryo-EM) revealed homogenous spherical nanoparticles (NPs) dispersion with little aggregation (Figure 1a–c). Quantitative particle size analysis of SAAP-148 NPs (Figure 1b) revealed a mean diameter of 95 ± 23 nm (Figure 1d), with a polydispersity index (PDI) of 0.08 ± 0.1, a surface charge of 1.65 ± 0.1 mV, and with a loading capacity (*w*/*w*) of 0.44 % (EE = 86.7 ± 0.3%). Halicin NPs (Figure 1c) are 118 ± 31 nm in diameter with a PDI of 0.20 and a surface net charge of −1.2 ± 0.2 mV. The blank NPs (Figure 1a) revealed a diameter of 97 ± 25 nm with a PDI of 0.20 and a surface charge of −3.26 ± 0.4 mV. SAAP-148 NPs exhibited a biphasic release profile in which 50% of the peptide was released after 5 h, followed by sustained release, for up to 21 days, of the remaining 50% of the encapsulated peptide (Figure 1e). Moreover, 100% of the SAAP-148 solution was completely dialyzed within 3 h under the same conditions. Fitting the data for the release of peptide from NPs, according to the Korsmeyer–Peppas Model (Figure 1f), resulted in a release exponent value (*n*) of 0.99, which correlates with non-Fickian zero-order peptide release.

### 2.2. Antibacterial Activities of SAAP-148 NPs

#### 2.2.1. In Vitro Killing Activity

Firstly, we investigated the antimicrobial activity of SAAP-148 NPs and free peptides against planktonic bacteria consisting of AMR bacteria, namely *S. aureus* and *A. baumannii* (Table 1). Antimicrobial data revealed that SAAP-148 NPs reached the lethal concentration to kill 99.9% (LC_99.9_) of planktonic *S. aureus* and *A. baumannii* at 12.8 µM and 1.6 µM after 24 h, respectively. Moreover, SAAP-148 solution reached the LC_99.9_ between 1.6–3.2 µM and ≤0.8 µM against planktonic *S. aureus* and *A. baumannii* after 24 h, respectively (Table 1). Expectedly, blank NPs did not induce any antimicrobial activity against any of the bacterial strains used in this study. In addition, SAAP-148 NPs induced a dose-dependent inhibition of planktonic AMR *S. aureus* and *A. baumannii* after 4 h, and they maintained this level of inhibition after 24 h (Supplementary Material Figures S1 and S2). Together, this data indicates that SAAP-148 NPs are significantly less effective in the killing of planktonic AMR bacteria compared to the peptide solution.

#### 2.2.2. In Vitro Anti-Biofilm Activity

Next, we determined antibiofilm efficacy of SAAP-148 NPs against AMR *S. aureus* and *A. baumannii* biofilms that were generated during a period of 24 h (Table 1). Data revealed that the exposure time of SAAP-148 NPs is directly proportional to increasing antimicrobial activity against biofilms of both strains. For instance, the biofilm inhibitory concentration (BIC_99.9_) against *S. aureus* biofilms of SAAP-148 NPs decreased from >204.8 µM at 4 h to 204.8 µM at 72 h. Similarly, regarding *A. baumannii* biofilms, the BIC_99.9_ concentration of SAAP-148 NPs decreased from 204.8 µM at 4 h to 51.2 µM at 72 h. The BIC_99.9_ concentrations for SAAP-148, in solution, was 25.6 µM regarding both bacterial biofilms for up to 24 h, with no improvement in killing activity at longer exposure times. As before, blank NPs did not exhibit any antibiofilm activity at any of the time-points tested. Together, the in vitro antibiofilm data suggests that increasing SAAP-148 NPs exposure time positively correlates with improved antibiofilm activity.

### 2.3. Cytotoxicity and Hemolytic Activity of SAAP-148 NPs

#### Cytotoxicity against Human Skin Fibroblasts

**Figure 2:**
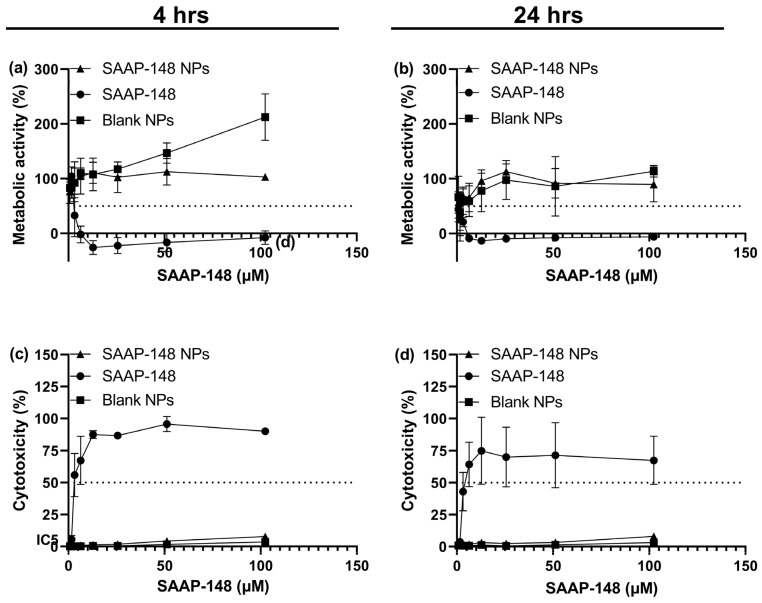
Cytotoxicity upon exposure of human skin fibroblasts monoculture to SAAP-148 NPs (triangles), SAAP-148 solution (circles), and blank NPs (squares). Metabolic activity based on water-soluble tetrazolium salt (WST-1) assay after (**a**) 4 h and (**b**) 24 h. Cytotoxicity against human skin fibroblasts, after (**c**) 4 h and (**d**) 24 h, based on lactate dehydrogenase (LDH) release. Metabolic activity and cytotoxicity assays were both performed in 0.5% human serum medium at 37 °C, with 95% humidity and 5% CO_2_. Data are retrieved from the mean of three biological replicates performed with three technical duplicates (*n* = 3). Inhibitory concentration (IC_50_) indicates the inhibitory concentration to induce 50% cytotoxicity.

**Figure 3:**
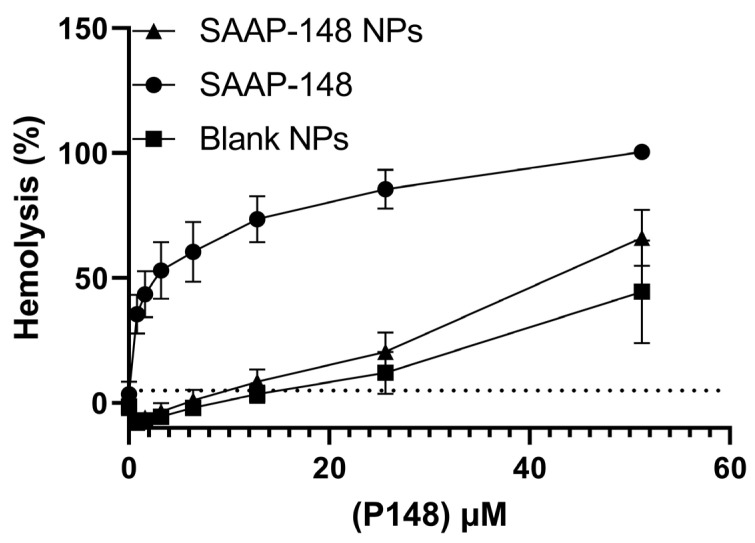
Hemolytic activity of SAAP-148 NPs (triangles) compared to SAAP-148 solution (circles) and blank PLGA NPs (squares) in PBS after 1 h. Hemolytic activity is based on 5% hemolysis of human erythrocytes in PBS, after 1 h incubation at 37 °C, with 95% humidity and 5% CO_2_. Data are retrieved from the mean of three biological replicates performed with three technical duplicates (*n* = 3). Hemolytic activity is based on 5% hemolysis of human erythrocytes.

Subsequently, we evaluated the cytotoxic and hemolytic activities of SAAP-148 NPs compared to free peptide (Figure 2a–d). Results showed that the encapsulation of SAAP-148 in PLGA NPs decreased the peptide’s cytotoxicity by at least 41-fold and 24-fold against the primary skin fibroblasts after 4 and 24 h, respectively (Table S1). The effect of the metabolic activity of human skin fibroblasts was also improved by ≥37-fold and ≥128-fold, following exposure to SAAP-148 NPs, after 4 and 24 h, respectively. In agreement, encapsulation of peptide decreased the hemolytic activity by at least 10-fold against isolated human erythrocytes in PBS (Figure 3) Collectively, these results indicate that the encapsulation of SAAP-148 in PLGA NPs drastically decreased the cytotoxic and hemolytic activities of this peptide.

### 2.4. Selectivity Index of SAAP-148 NPs

**Figure 4:**
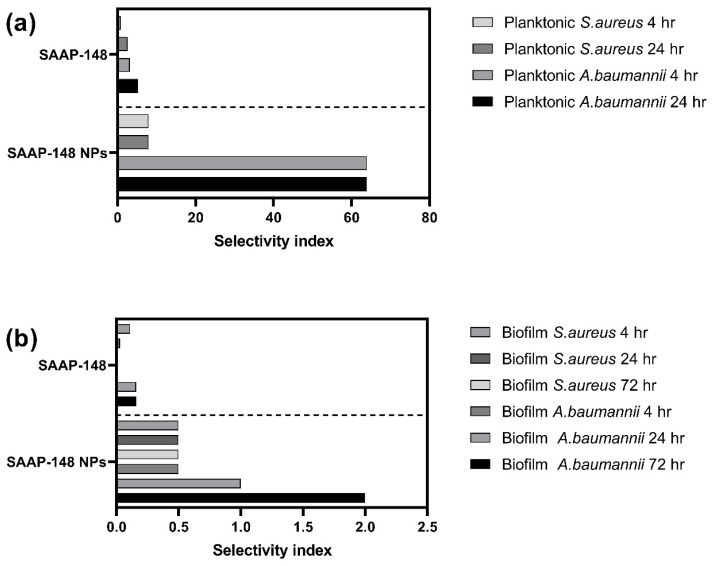
Selectivity index of SAAP-148 NPs and SAAP-148 solution based on the killing of (**a**) planktonic and (**b**) biofilms composed of AMR *S. aureus* and *A. baumannii* bacteria compared to the cytotoxicity against human skin fibroblasts. The selectivity index was calculated by dividing the median cytotoxicity value (IC_50_) obtained from human primary skin fibroblasts by the median antimicrobial value (LC_99.9_) obtained for each time point against *S. aureus* or *A. baumannii* (IC_50_/ LC_99.9_). The data used to calculate the selectivity index was extracted from the median of three independent cytotoxicity and antibacterial activity experiments, each performed in triplicate.

Subsequently, we calculated the selectivity index of SAAP-148 NPs and free peptide based on the ratio between cytotoxicity and antimicrobial activity (IC_50_/LC_99_) (Figure 4). Data showed that the selectivity index of the SAAP-148 NPs was increased by 10-folds after 4 h incubation against *S. aureus*, and increasing the exposure time to 24 h induced a 3-fold increase compared to the free peptide. Similarly, the selectivity index of SAAP-148 NPs against planktonic *A. baumannii* improved by 20-fold after 4 h, and increasing the exposure time to 24 h induced a 12-fold increase compared to the free peptide. Furthermore, increasing the exposure time of SAAP-148 NPs significantly increased the selectivity index against *S. aureus* and *A. baumannii* biofilms compared to free peptide. More specifically, increasing the exposure time from 4 h to 72 h increased the selectivity index by almost 17-fold against *S. aureus* biofilm and by up to 12-fold against *A. baumannii* biofilm compared to the free peptide. Together, these results indicate that encapsulation of SAAP-148 in PLGA NPs substantially increased the selectivity index of the peptide.

### 2.5. 3D Human Skin Equivalents (HSEs

#### 2.5.1. In Vitro Antimicrobial Activity in 3D HSEs

**Figure 5:**
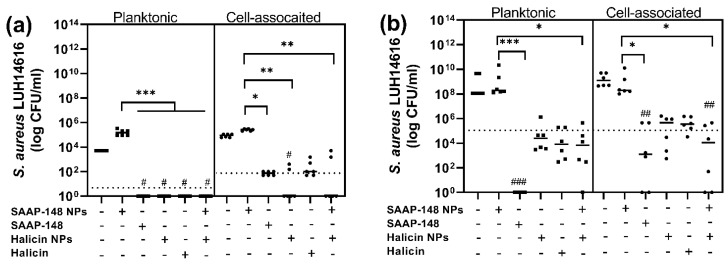
Bactericidal activity against planktonic (squares) and cell-associated (circles) AMR *S. aureus* in 3D human skin equivalents (HSE) after exposure for (**a**) 4 h and (**b**) 24 h. Data are presented as the median of three independent experiments performed in duplicate. The average bacterial concentration, at which 99.9% of bacteria are killed (LC_99.9_), is represented by the dashed lined. Statistical significance compared to PBS (negative control) is depicted with a number sign (#) and compared to SAAP-148 NPs is depicted with an asterisk (*) above each data set where a *p* ≤ 0.05 is considered statistically significant, as calculated by the Kruskal–Wallis test with Dunn’s post-hoc test. Statistical significance between two groups is depicted as */# for *p* ≤ 0.05, **/## for *p* ≤ 0.01, or ***/### for *p* ≤ 0.001. SAAP-148 concentration = 204.8 µM; halicin concentration = 409.6 µM.

Subsequently, we studied the efficacy of SAAP-148 NPs in 3D HSE models infected with AMR *S. aureus*. Antibacterial data from the 3D HSEs showed that SAAP-148 NPs were significantly less effective in eliminating planktonic and cell-associated AMR *S. aureus* than the peptide solution. Hence, a dual drug delivery strategy was implemented, consisting of combined SAAP-148 NPs and halicin NPs as one treatment (Figure 5). The data showed that SAAP-148 NPs and halicin NPs induced an improved antimicrobial response compared to SAAP-148 NPs alone against planktonic (*p* = 0.0005) and cell-associated bacteria (*p* = 0.0024), whereas their efficacy was similar to that of halicin NPs alone (*p* > 0.05) after 4 h (Figure 5a). After 24 h exposure, similar data were obtained regarding planktonic and cell-associated bacteria (Figure 5b). Moreover, analysis of the data showed that the combined SAAP-148 NPs and halicin NPs induced a statistically significant reduction in cell-associated *S. aureus* compared PBS (*p* = 0.0018), while observed changes were not statistically significant for either SAAP-148 NPs (*p* = 0.99) or halicin NPs alone (*p* = 0.08) after 24 h. Interestingly, the data showed that halicin NPs exhibited a similar antimicrobial activity compared to the halicin solution against planktonic or cell-associated bacteria after 4 or 24 h (*p* > 0.05 for all). Together, the data indicate that SAAP-148 NPs and halicin NPs, as well as halicin NPs alone, but not SAAP-148 NPs alone, are effective against planktonic and cell-associated bacteria in the 3D HSEs.

#### 2.5.2. Colocalization of SAAP-148 NPs with Bacteria in 3D HSEs

**Figure 6:**
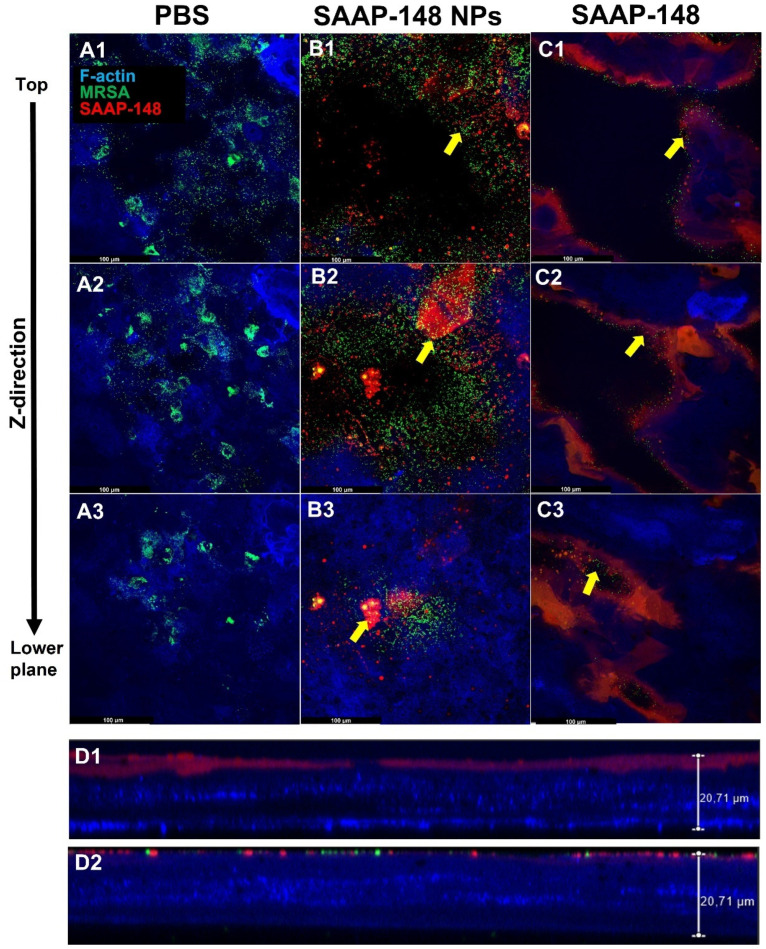
Representative confocal micrographs, taken across the Z-axis of 3D human skin equivalents (HSEs), are fluorescently labeled with Phalloidin (blue), infected for 1 h with GFP-labeled MRSA (green), and then, subsequently exposed for 24 h to (**A1**–**A3**) PBS, (**B1**–**B3**) 20 µM carboxytetramethylrhodamine (TAMRA)-labeled SAAP-148 NPs, or (**C1**–**C3**) 20 µM TAMRA-labeled SAAP-148 solution (red). Scale bar: 100 µM. Side view of (**D1**) SAAP-148 solution, and (**D2**) SAAP-148 treated 3D HSEs (thickness = ~20 µm). Yellow arrows indicate colocalization of SAAP-148 NPs/SAAP-148 solution with bacteria on the top layer of the HSEs. Representative images are of observations from *n* = 3 independent experiments.

Lastly, we investigated the colocalization of the fluorescently labeled SAAP-148 NPs or SAAP-148 solution with green fluorescent protein (GFP)-labeled MRSA in 3D HSEs (Figure 6). Confocal micrographs (Figure 6A1–C3) were obtained starting from the top layers of the skin models perpendicular to the plane of the cell midpoint (z-axis). Confocal micrographs obtained from 3D HSEs revealed cell-associated bacteria with the top layers of the skin models after 24 h (Figure 6A1–A3). Moreover, the epidermal models revealed that SAAP-148 NPs (Figure 6B1–B3) and SAAP-148 solution (Figure 6C1–C3) were localized in proximity to bacteria, with the free peptide exhibiting a noticeable reduction in GFP-MRSA. Furthermore, cross-sectional micrographs further confirmed the colocalization of SAAP-148 NPs and SAAP-148 solution with bacteria in the top layers of the epidermal models (Figure 6D1,D2). Altogether, confocal analysis of the micrographs indicates colocalization of SAAP-148 NPs with bacteria on the surface of the skin models after 24 h.

## 3. Discussion

The aim of this study was to enhance the selectivity index of SAAP-148 by formulating this peptide in PLGA NPs. The selectivity index is typically used as a pre-clinical screening strategy by determining the ratio of toxic concentration by its effective therapeutic concentration [15,16]. Previous studies have shown that a higher selectivity index is an early indicator of drug success and tends to decrease as it progresses through the development pipeline [17,18]. Although the selectivity index does not allow the extrapolation of doses administered in vivo [19], it is a relevant indicator to use in the screening procedure for further evaluation. Previous studies showed that the encapsulation of AMPs—physiochemically similar to SAAP-148—in PLGA NPs significantly decreased the peptide’s cytotoxicity and increased its stability [13,20]. Based on these observations, we hypothesized that encapsulation of SAAP-148 in PLGA NPs would enhance the peptide’s selectivity index by improving its stability and inducing controlled release of peptide.

An important functional characteristic of PLGA NPs pertains to its in vitro physiochemical properties, including particle size, surface area, surface chemistry, and release profile. Physiochemical analysis of SAAP-148 NPs revealed a uniform particle distribution with controlled release of peptide over 21 days. Generally, smaller sized nanoparticles (<200 nm) elicit higher antimicrobial activity due to a larger surface area-to-mass ratio, resulting in higher interactions on bacterial surfaces [21]. Furthermore, the release profile of peptide revealed a biphasic pattern consisting of a burst phase after several hours, followed by a sustained release phase lasting several days. Fitting the release profile into the Korsmeyer–Peppas model suggested that the release of peptide was governed by swelling and/or polymer chain relaxation [22,23]. This indicated that peptide release from PLGA NPs is only a function of time regardless of peptide concentration [24]. The higher amount of peptide released during the burst phase is beneficial, as it guarantees that SAAP-148 is rapidly bioavailable to directly interact with bacteria to induce membrane destabilization, whilst the sustained release phase maintains adequate peptide levels lasting for several days. Meanwhile, all the free peptide is directly bioavailable, resulting in greater cytotoxicity to mammalian cells.

Comparing the efficacy of SAAP-148 NPs and this peptide in solution revealed a reduction in antimicrobial and antibiofilm activities of the peptide in NPs against AMR *A. baumannii* and *S. aureus*. Interestingly, the SAAP-148 NPs demonstrated a higher antibiofilm activity after 72 h, as compared to 4 h, against AMR *S. aureus* and *A. baumannii*, suggesting a dose-response relationship based on the in vitro release data. Impressively, the cytotoxicity of SAAP-148 NPs against human skin fibroblasts and erythrocytes was significantly reduced compared to SAAP-148 solution. This data indicates that the NPs are very effective in shielding this peptide’s cytotoxic activities to mammalian cells by reducing the electrostatic interactions to the membrane surface [25]. Combined, these results indicate that SAAP-148 NPs exhibited a significantly higher selectivity index compared to free peptide.

Furthermore, previous data from our laboratory showed that SAAP-148 and halicin induced a synergistic response against AMR planktonic and biofilm bacteria, and these favorable interactions were confirmed for AMR *S. aureus* infected 3D HSEs [26]. However, synergism was not confirmed in PLGA-formulated SAAP-148 and halicin NPs. To fill this knowledge gap, and because SAAP-148 NPs alone were poorly effective in eliminating bacteria from such models, we tested whether synergism could also be attained with the PLGA NP-formulated counterparts of SAAP-148 and halicin. For this reason, 3D HSEs infected with AMR *S. aureus* were exposed to combined SAAP-148 NPs and halicin NPs to evaluate the potential synergism in this model and compare them against their respective counterparts. Halicin is a powerful antibiotic that was identified by a machine-learning algorithm using artificial intelligence, and it has low toxicity to human cells [27]. Halicin is a c-Jun N-terminal protein kinase (JNK) inhibitor, which disrupts the electrochemical gradient of bacteria necessary for growth [28]. Combined, SAAP-148 NPs and halicin NPs displayed better antibacterial activity than SAAP-148 NPs alone but showed similar antibacterial activity as halicin NPs alone against planktonic and cell-associated *S. aureus* in 3D HSE models. It is possible that differences in release of SAAP-148 and halicin from the NPs could explain the absence of synergism between these antibacterial agents at 24 h. Using a fast-release drug delivery system for the delivery of SAAP-148, such as hyaluronic acid nanogels, could improve the therapeutic activity and attain synergism in this setup [29]. Nevertheless, combined, SAAP-148 NPs and halicin NPs were slightly more effective in eradicating cell-associated *S. aureus* than either SAAP-148 NPs or halicin NPs alone after 24 h. Notably, halicin NPs exhibited a similar antibacterial activity as halicin in solution, suggesting that the physiochemical properties of the encapsulated biomolecule could play a crucial role in determining its antibacterial activity when encapsulated in NPs.

Subsequently, we compared the localization pattern of fluorescently labeled SAAP-148 NPs or peptide solution with GFP-MRSA in the 3D HSE models. Confocal micrographs revealed localization of SAAP-148 NPs and SAAP-148 solution within the proximity of bacteria predominantly situated in the top layers of the epidermal models. Cross-sectional micrographs revealed that SAAP-148 in solution associated better with keratinocytes than SAAP-148 NPs, which was expected since peptide in solution is more readily taken up by the keratinocytes compared to slow releasing peptide NPs [30]. A possible explanation for the formulated peptide’s limited efficacy in the skin models is the anionic surface charge of NPs. Previous studies showed that anionic NPs elicit an electrostatic barrier, which limits their adherence to the surface of negatively charged mammalian cells [31,32,33]. Hence, cationic NPs may overcome the initial repulsion forces with the keratinocytes and enhance peptide release into the extracellular milieu [34]: for instance, by incorporating charged groups (amino or carboxyl) into a polymer backbone or by coating the NPs with positively charged polymers/proteins (e.g., polyethylenimine, bovine serum albumin) [35]. However, cationic NPs are limited by their cytotoxicity due to greater plasma membrane destabilization than their anionic counterparts [36]. Hence, further research is needed to fully study the influence of surface charge in relation to the biological effects.

To further improve the efficacy of SAAP-148 NPs, surface functionalization to specifically target bacteria, most specifically biofilms, could be considered. For example, Eissa et al. (2016) reported on carbohydrate/glycan functionalized polymeric NPs that prevented bacterial adhesion by blocking carbohydrate structures on mammalian cell surface [37]. Tan et al. (2022) synthesized chitosan nanoparticles that were functionalized with β-1,3-glucanase to degrade the *Candida albicans* biofilm matrix, which is fundamental for microbial growth and drug resistance [38]. Ivanova et al. (2018) functionalized inert poly(methyl vinyl ether/maleic) acid NPs with the antimicrobial aminocellulose and antifouling hyaluronic acid using layer-by-layer coating technology [39]. Furthermore, Ucak et al. (2020) showed that aptamer-functionalized vancomycin-encapsulated PLGA NPs bind selectively to bacterial cell surface antigens [40,41]. Alternatively, NPs could be functionalized to target (infected) host cells. For instance, PLGA fibers cross-linked to collagen showed enhanced skin cell adhesion by promoting integration [42]. PLGA conjugated to transferrin were shown to selectively target transferrin receptors, which are highly expressed by keratinocytes [43]. To this end, surface functionalization of PLGA NPs presents a promising way to specifically target bacterial biofilms and/or further enhance their association with colonized/infected host cells

## 4. Materials and Methods

### 4.1. Materials

All chemicals were used without further purification. SAAP-148 (Ac-LKRVWKRVFKLLKRYWRQLKKPVR-NH_2_) MW 3265.06 Da, >90% purity, synthesis, purification and identification were conducted according to a similar procedure as has been described before by Nell et al. (2006) [44]. Fluorophore-labeled peptide, TAMRA-SAAP-148, was used at 96.9% purity and MW of 3679.7 Da and sequence AC-LK(6-TAMRA)RVWKRVFKLLKRYWRQLKKPVR-NH2 was purchased from Pepscan (Lelystad, The Netherlands). Lyophilized SAAP-148 was dissolved and diluted in phosphate-buffered saline (PBS), pH 7.4, 140 mM NaCl (Fresenius Kabi Nederland B.V., Huis ter Heide, The Netherlands), and stored in aliquots at −20 °C until use. Poly(lactide-co-glycolic) acid (PLGA) [acid-terminated, 5002A, 50:50 lactide: glycolide ratio, intrinsic viscosity 0.2 dL/g], ethylacetate, human serum, tetracycline, Triton X-100, and cell proliferation reagent WST-1 were obtained from Sigma-Aldrich (St. Louis, MO, USA). Polyvinyl alcohol (Mw: 20,000–30,000), tryptone soy broth (TSB), and brain heart infusion broth (BHI) were obtained. Additionally, 15 mL and 50 mL cellstar^®^ polypropylene tubes, 96-wells round bottom polystyrene plates, microplate lid polystyrene, polypropylene 1.5 mL eppendorf tubes, and disposable inoculating loops were all sterile and purchased from Greiner Bio-One (Kremsmünster, Austria). Prolong diamond antifade mountant, human recombinant epidermal growth factor (EGF), Bovine Pituitary Extract (BPE), fetal calve serum (FCS), and penicillin 10,000 U/mL and streptomycin 10,000 U/mL (P/S), as well as DMEM, Ham F12 medium, and Alexa Fluor™ 405 Phalloidin were purchased from ThermoFischer Scientific. Blood agar plates (Trypcase Soy Agar plates + 5% sheep blood) were purchased from Biomerieux (Marcy-l’Étoile, France). Incubator shaker series, Innova 40, was purchased from New Brunswick Scientific (Edison, NJ, USA), Centrifuge Beun de Ronde STEK krt7276), CO_2_ incubator Hereus Hera cell 240, was purchased from Thermo Electron Corporation (Waltham, MA, USA). LDH cytotoxicity detection kit and Bovine serum albumin (BSA) were purchased from Roche Diagnostics (Basel, Switzerland), and dialysis membranes (Spectra-Por^®^ Float-A-Lyzer^®^ membrane G2 with MWCO of 100 kD Spectrum labs) were obtained Merck & Co. (Rahway, NJ, USA). Pooled human plasma was obtained from Sanquin (Leiden, The Netherlands).

### 4.2. Preparation of PLGA NPs

PLGA NPs were formulated using the solvent evaporation double emulsion water-in-oil-in-water (W/O/W) technique using a previously described method [45,46]. Briefly, 50 mg of PLGA was dissolved in 1.5 mL of ethyl acetate. SAAP-148 was dissolved in Milli-Q water at a concentration of 2 mg/mL. Additionally, 0.5 mL of SAAP-148 2 mg/mL solution was added dropwise while vortexing at 1000 rpm to the dissolved PLGA, and then, it was sonicated at 25% amplitude for 1 min (20 s on/off cycle) on ice water to form the primary emulsion. Subsequently, 2.5 mL of PVA solution (25 mg/mL) was added dropwise to the primary emulsion while vortexing at 1000 rpm, and then, it was sonicated again at the same setting. Organic solvent was removed by evaporation using a rotatory evaporator for 1 h at 100 mbar at room temperature (RT). The formulated NPs were then washed in Milli-Q and lyophilized overnight before storage at −20 °C.

### 4.3. Peptide Analysis with Ultra-High Performance Liquid Chromatography

SAAP-148 was detected by ultra-high performance liquid chromatography (UPLC, Waters, Milford, MA, USA) equipped with a BEH C_18_, 1.7 µM, 130 Å, 2.1 × 100 mm column over 8 min with a linear gradient of two buffers: (A) Milli-Q and (B) acetonitrile, both containing 0.05% trifluoroacetic acid. Starting at a ratio of 95% A and 5% B to 25% A and 75% B and ending after 8 min at a flow rate of 0.5 mL/min and UV detection of 214 nm, data were analyzed using Mass-Lynx Software V4.2.

### 4.4. Characterization of SAAP-148 NPs

#### 4.4.1. Encapsulation Efficiency and Drug Loading

The amount of encapsulated peptide was calculated by measuring the amount of free peptide using an indirect method adapted from the literature [47]. After lyophilization, the SAAP-148 NPs were suspended in 1 mL of PBS in 2 mL microtube, and then, they were mixed by vortexing for 10 min to extract the free peptide in the aqueous phase. Subsequently, the suspension was centrifuged under 10,000 rpm for 30 min at RT to separate the polymeric debris and the amount of free peptide was measured in the supernatant with UPLC, as previously described (Section 4.3).

The encapsulation efficiency (EE) of SAAP-148 was calculated by the following equation:

$$\%\mathrm{EE}:\frac{Total\;amount\;of\;peptide\;\left(\mu g\right)-amount\;of\;peptide\;in\;the\;supernantant\left(\mu g\right)}{Total\;amount\;of\;peptide\;\left(\mu g\right)}\times 100$$

The loading capacity was similarly calculated:

$$\%\mathrm{LC}:\frac{Total\;amount\;of\;peptide-amount\;of\;peptide\;in\;the\;supernantant}{Total\;weight\;of\;SAAP-148\;NPs\;\left(\mu g\right)}\times 100$$

#### 4.4.2. Drug Release

The in vitro release profile of SAAP-148 from PLGA NPs was analyzed with SpectraPor^®^ Float-A-Lyzer^®^ membrane G2 dialysis device with MWCO of 100 kD. The membranes were soaked in Milli-Q before adding 10% ethanol to remove present salts in the dialysis instrument. The membranes were then washed with Milli-Q before coating with 1 mg/mL lysozyme, for 1 h, while stirring at 37 °C. The lysozyme was then removed, and the membrane was washed again with Milli-Q. Then, 1 mL of 100 µM SAAP-148 NPs were added to the inner well, and 5 mL of PBS was added to the outer well. At each time-point, 200 µL of fluid was removed from the outer compartment and replaced with fresh PBS of the same volume. All samples were stored at −20 °C until further analysis with UHPLC.

#### 4.4.3. Cryo Electron Microscopy (cryo-EM)

The 300 mesh EM grids (Quantifoil R2/2, Jena, Germany) were glow-discharged by 0.2 mbar air for two minutes using the glow discharger unit of an EMITECH K950X. The 3 µL of the sample was applied per glow-discharged grid, and the grid was vitrified using an EMGP (Leica, Wetzlar, Germany) at room temperature and 100% humidity. For vitrification, excess sample was removed by blotting for one second to Whatman #1 filter paper directly followed by plunging the grid into liquid ethane (−183 °C), after which the grid was stored under liquid nitrogen until further use. Cryo EM imaging was performed at 120 kV on a Tecnai 12 electron microscope (FEI Company, Eindhoven, The Netherlands) after mounting the grid in a Gatan 626 cryo-holder. A 4 k × 4 k Eagle camera (FEI Company) was used to record images with focus between 5–10 µm and an 18,000× magnification (pixel size 1.2 nm).

### 4.5. Antibacterial and Anti-Biofilm Activities of SAAP-148 NPs

#### 4.5.1. Antibacterial Killing Studies

Colonies of Gram negative (*A. baumannii* RUH875) and Gram positive (methicillin resistant *S. aureus* LUH14616; NCCB100829) were incubated for 2.5 h in TSB to mid-logarithmic phase. The mid-logarithmic phase bacteria were washed and suspended in PBS before measuring the optical density at 600 nm with a spectrometer. Bacteria were then diluted in PBS to a concentration of 5 × 10^6^ colony-forming units (CFU)/mL and kept on ice. To each 96-well, 30 µL of SAAP-148, SAAP-148 NP, or blank PLGA NPs was added. To make a final plasma concentration of 50%, 50 µL/well of human plasma was added, followed by 20 µL of bacterial suspension previously prepared. The plate was then covered with a plastic sealer and lid and mixed for 10 s using a plate shaker, and it was centrifuged for 1 min at 12,000 rom at RT. The plate was then incubated for 4 or 24 h at 37 °C. Each well was then diluted 10× in PBS and plated on a Mueller–Hinton agar plate. Then, they were incubated overnight at 37 °C. The bacterial colonies were manually counted and converted to CFU/mL. All bacterial studies performed under sterile conditions.

#### 4.5.2. Anti-Biofilm Studies

Bacteria were grown to the mid-logarithmic phase in brain heart infusion broth (BHI). Then, 100 µL of 1 × 10^7^ CFU/mL in BHI was added in a number of wells of a flat-bottom polypropylene plate. The plates were covered with breathable seals and incubated for 24 h at 37 °C in a humidified controlled environment. The bacterial suspension was then discarded, and the wells were washed 2 × 100 µL PBS before treatment with peptide. The plate was then covered with a sealer and incubated for either 4, 24, or 72 h at 37 °C under the same humidified environment. The treatment wells were then washed twice with 100 µL PBS, covered with an aluminum seal, and placed in a waterproof plastic bag before sonicating for 10 min to dislodge the biofilm. To further disrupt the biofilm, vigorous pipetting was introduced. The biofilm colonies were diluted and plated on a Mueller–Hinton plate and converted to CFU/mL.

### 4.6. Cytotoxic Activities of SAAP-148 NPs

Human skin fibroblasts (HSFs) were cultured in culture flasks and sustained with DMEM supplemented with 1% (*v*/*v*) GlutaMAX™, 1% (*v*/*v*) P/S, and 5% (*v*/*v*) FCS. HSFs were seeded at a density of 20,000 cells in DMEM medium, supplemented with 1% P/S and 0.5% human serum, and incubated overnight at 37 °C at 5% CO_2_. Peptide dilutions were made in the DMEM medium supplemented with 1% P/S and 0.5% human serum. Additionally, 75 µL of the medium from the wells were removed before adding 25 µL of peptide dilutions to the remaining 25 µL medium in the wells. As a negative control, 25 µL medium without peptide was used. As a positive control, 25 µL of 1% Triton in the medium was used. The treated cells were returned to the incubator for either 4 or 24 h. At the end of the incubation period, 25 µL of the supernatant was added to a new round bottom 96-well plate to measure LDH release.

#### 4.6.1. Cytotoxicity Based on LDH Release

The collected supernatant (or subnatants from 3D human skin equivalents; Supplementary Material, Figure S3) was centrifuged at 2000 rpm for 3 min and diluted 1:10, in PBS, in a 96-well flat bottom plate. The LDH reaction mix was added at 50 µL per well according to the manufacturer’s instructions, the diluted plate was incubated at RT for 30 min, and then, the absorbance was read at 490 nm and 600 nm as a reference.

#### 4.6.2. Cell Metabolism Based on WST-1

To the remaining cells, 20 µL of fresh medium, supplemented with 0.5% human serum, was added per well. Then, 5 µL of WST reagent was added according to the manufacturer’s instructions. The cells were incubated at 37 °C, for 30–60 min, until a color change could be observed. The absorption was measured at 440 nm, and 590 nm was used a reference wavelength. The data provided is based on % of the positive control (LDH: Triton X-100100, WST-1: PBS) calculated using the following formula:

$$\%\;Cytotoxicity\;or\;metabolic\;activity=\frac{test\;sample-negative\;control}{positive\;control-negative\;control}\times 100$$

#### 4.6.3. Hemolytic Activity

Whole blood was obtained from healthy volunteers in citrate tubes and spun for 10 min at 3000 rom to pellet the erythrocytes (Sanquin, NVTO128.02, with informed consent). The erythrocyte pellet was then transferred to a plastic 15 mL tube, and the cells were washed 3× with PBS by vortexing, then spinning at 1000 rpm, and then discarding the supernatant. The erythrocyte suspension was then diluted to 20% in PBS and, then, in PBS to a 2% erythrocyte suspension. Stock peptide and SAAP-148 NPs solutions were diluted separately to their respective concentrations in Milli-Q, using a 96-well polypropylene V-bottom plate, to a final volume of 25 µL. As positive control, 25 µL of Triton-X was used instead. Then, 50 µL of PBS was added, followed by 25 µL of the 2% erythrocyte suspension, and the plate was covered with a lid and mixed by shaking for 10 s at 300 rpm before incubation at 1 h at 37 °C. Subsequently, the plan was spun for 3 min at 1200 rpm, and 85 µL of the supernatant was transferred to a 96-well flat-bottom plate to measure the optical density at 415 nm. The following formula was used to determine the % hemolysis based on the manufacturer’s instructions: IC_50_ LC_99.9_

$$\%\;hemolysis=\frac{test\;sample-negative\;control}{positive\;control-negative\;control}\times 100$$

#### 4.6.4. Selectivity Index

The selectivity index of the peptide was calculated by taking the ratio between cytotoxicity using LDH release and antibacterial activity using the following formula:

$$Selectivity\;index=\frac{Cytotoxicity\;\left({\mathrm{IC}}_{50}\right)}{Antibacterial\;activity\;\left({\mathrm{LC}}_{99.9}\;or\;BIC_{99.9}\right)}$$

### 4.7. Experiments with 3D Human Skin Equivalents (HSE)

#### 4.7.1. Culturing of Ker-CT Keratinocytes

Human skin equivalents (HSEs) were cultured as previously described in detail [48]. In brief, human keratinocytes of the Ker-CT cell line (ATCC^®^ CRL-4049™) were cultured until 70–80% confluency in keratinocyte serum-free medium supplemented with L-Glutamine supplemented with bovine pituitary extract, human recombinant epidermal growth factor, 1% (*v*/*v*) penicillin-streptomycin (ThermoFischer Scientific; Waltham, MA, USA), and calcium chloride (CaCl_2_; Merck, Kenilworth, NJ, USA). The keratinocytes were harvested using 0.05% trypsin-EDTA (ThermoFischer Scientific), neutralized with stop solution (10% FCS), and washed. On day 0, the HSEs were grown by seeding 500 µL of 5 × 10^5^ cells/mL Ker-CT suspension diluted in DermaLife K medium (Lifeline Cell Technology; San Diego, CA, USA) into each transwell insert [pore size 0.4 µm in 12-well plates (Greiner Bio-One)] and below the well. On day 2, the medium above and below the insert was replenished with K0/CNT/1:1:1 cell culture medium, consisting of 50% K0 medium (3 part DMEM and 1 part Ham’s F12) supplemented with 1% P/S, 200 ng/mL hydrocortisone, 2 µM isoproterenol, 500 ng/mL isoproterenol, 500 ng/mL bovine insulin, 53 nM selenious acid, 10 mM L-serine, 20 µM L-carnitine (ThermoFischer Scientifics), and 50% CNT-Prime 3D Barrier Medium (CellnTEC; Bern, Switzerland) supplemented with a 1:1:1 lipid mixture consisting of 15 µM linoleic acid, 25 µM palmitic acid, and 7 µM arachidonic acid (ThermoFischer Scientific) in 80 mg/mL bovine serum albumin (Roche Diagnostics; Basel, Switzerland). On day 3, the models were air exposed by removing the supernatant on top of the insert. On day 4, and from here on, the medium from the bottom well was replenished every 2 days with K0/CNT/2:1:1 medium, as mentioned above, supplemented with 2:1:1 lipid mixture, consisting of 30 µM linoleic acid, 25 µM palmitic acid, and 7 µM arachidonic acid in 80 mg/mL bovine serum albumin, until the skin models matured. On day 14, the mature epidermal models were suspended in K0/CNT/2:1:1 medium, without P/S, for 48 h before conducting antibacterial testing with peptides.

#### 4.7.2. Infection and Treatment of 3D HSE Models

GFP-MRSA (USA300 JE2) was transferred from the glycerol stock to TSB containing 50 µg/mL tetracycline for overnight culture at 37 °C. The next day, the bacterial culture was split and grown for an additional 2.5 h, to the mid-log phase at 37 °C, before washing and optical density measurement. On the day of the infection, any residual liquid above the model was removed. The models were then infected by adding 300 µL of 3.3 × 10^5^ CFU/mL AMR *S. aureus* (10^5^ CFU/model) or GFP-MRSA for confocal microscopy experiments and incubated for 1 h at 37 °C. As a control, the inoculum was diluted and counted. After 1 h, the supernatant was removed, and the models were washed 1× with 500 µL PBS. Then, two models were retained to measure the starting planktonic and cell-associated bacterial concentration after 1 h (T0). Thereafter, 300 µL of the desired peptide solution (plain or fluorescently labeled peptide) was added to the model and incubated for 24 h at 37 °C and 5% CO_2_. After 24 h of incubation, the supernatant was removed and stored for the counting of the planktonic fraction. The skin models were extracted by carefully removing the skin models from the inserts with a scalpel and transferring them to a potter system with 0.5 mL of PBS. To extract the cell-associated bacteria fraction, the models were homogenized with a bead-beater (Precellys 24 tissue homogenizer, Bertin Technologies, Montigny-le-Bretonneux, France) for 3 × 5000 rpm for 10 s with 10 s pause. The planktonic and cell-associated bacterial fractions that were separately diluted in PBS and CFU on MH agar plates were stored overnight in 37 °C overnight, and colonies were manually counted.

#### 4.7.3. Confocal Microscopy

The infected and treated 3D HSE models were washed with PBS before fixing with 1% paraformaldehyde (PFA), 500 µL apically and 1 mL basally, and incubated for 1 h at 4 °C. The models were washed with PBS and stored overnight in PBS at 4 °C. The skin models were, then, blocked with 1% BSA + 0.3% Triton X-100 in PBS (PBT) for 10 min at RT. The plastic inserts around the models were, then, carefully removed with a scalpel. The skin models were, then, stained with 50 µL of 1:50 diluted Alexa Fluor™ 405 dye Phalloidin (ThermoFischer Scientific) in PBT solution for 2 h at 4 °C to stain filamentous actin (F-actin) of the keratinocytes. The models were then washed 3× in PBS and 3× in Milli-Q before they were mounted on a glass microscope slide with the epidermis facing upwards. Diamond Antifade mountant was added to each sample, and a cover slip was placed on top. Mounted samples were kept at RT in the dark until imaging. All samples were imaged on a Leica SP8 up right confocal microscope, 3D images were produced using Leica SP8 WLL-2 inverted confocal microscope at 40× magnification, and images were process using LASX software version 3.7.6 (Leica Microsystems, Wetzlar, Germany).

### 4.8. Statistics

Statistical significance between groups was evaluated by the nonparametric Kruskal–Wallis test with Dunn’s post-hoc test with GraphPad Prism software version 9.3.1 (GraphPad Software, San Diego, CA, USA). Differences between groups were considered statistically significant at *p* ≤ 0.05.

## 5. Conclusions

To conclude, SAAP-148 NPs were successfully formulated with favorable physiochemical properties and the sustained release of peptide for up to 21 days. Combined studies of the antibacterial and cytotoxic activities of SAAP-148 NPs demonstrated higher selectivity index SAAP-148 in PLGA NPs compared to the free peptide. Antibiofilm studies indicated that SAAP-148 NPs exert optimal antibacterial activity after 72 h, suggesting a dose-response relationship, which corroborates the in vitro release data. Unfortunately, SAAP-148 NPs were not as effective as free peptide in eliminating bacteria from colonized 3D HSE, thus requiring further optimization of these NPs. Together, PLGA NPs present an interesting approach to further development as a treatment to combat AMR bacterial infections that require long-term sustained local release of peptide.

## 6. Patents

The SAAP-148 peptide is patented (WO2015/088344) by Leiden University Medical Center with co-inventors J.W.D. and P.H.N.

## Figures and Tables

**Table 1:** In vitro killing activity of formulated and unformulated peptides against planktonic and biofilm composed of AMR *S. aureus* and *A. baumannii* bacteria.

	AMR *S. aureus* LUH14616					AMR *A. baumannii* RUH875				
	LC99.9 (µM)		BIC99.9 (µM)			LC99.9 (µM)		BIC99.9 (µM)		
**Exposure**	**4 h**	**24 h**	**4 h**	**24 h**	**72 h**	**4 h**	**24 h**	**4 h**	**24 h**	**72 h**
**SAAP-148 NPs**	12.8 (1.6–12.8)	12.8 (6.4–12.8)	>204.8	>204.8	204.8	1.6 (0.8–1.6)	1.6	>204.8	102.4 (51.2–204.8)	51.2
**SAAP-148**	3.2 (0.8–6.4)	1.6 (0.8–1.6)	25.6 (25.6–102.4)	25.6	25.6	≤0.8	≤0.8	25.6 (25.6–51.2)	25.6 (25.6–102.4)	25.6
**Blank NPs**	>51.2	>51.2	>204.8	>204.8	>204.8	>51.2	>51.2	>204.8	>204.8	>204.8

In vitro killing results are expressed as the lethal concentration based on the minimum concentration at which 99.9% of the planktonic bacteria are killed compared to negative control (LC_99.9_). Antibiofilm activities are expressed as the lowest concentration of antimicrobial agents, resulting in a 99.9% reduction in bacteria in biofilms compared to control (BIC_99.9_). Results are provided as the median (and ranges where applicable) of three independent experiments, each performed in duplicates. If no range is indicated, the LC_99.9_ or BIC_99.9_ was identical in all experiments. Antibacterial studies against AMR planktonic *S. aureus* and *A. baumannii* were performed in 50% plasma (*v*/*v*) or PBS for the biofilms.

## Data Availability

The data presented in this study are available from the corresponding author upon request.

## Supplementary Materials

The following supporting information can be downloaded at: https://www.mdpi.com/article/10.3390/ijms24032867/s1.
