# Supplementary material for: Physical and Functional Characterization of PLGA Nanoparticles Containing the Antimicrobial Peptide SAAP-148
Source: Int J Mol Sci. 2023 Feb 2;24(3):2867. doi: 10.3390/ijms24032867 (PMC9918011; doi:10.3390/ijms24032867)

Supplementary material

Physical and functional characterization of PLGA nanoparticles containing the antimicrobial peptide SAAP-148

Figure S1. *In vitro* killing activities of SAAP-148-PLGA NPs (**a&d**), SAAP-148 (**b&e**), and PLGA NPs (**c&f**) against *S. aureus* LUH14616 after 4 hrs (**a-c**) and 24 hrs (**d-f**) of incubation in 50% plasma at 37°C. Dotted line represents the lethal concentration to kill 99.9% of bacterial counts ($LC_{99.9}$).

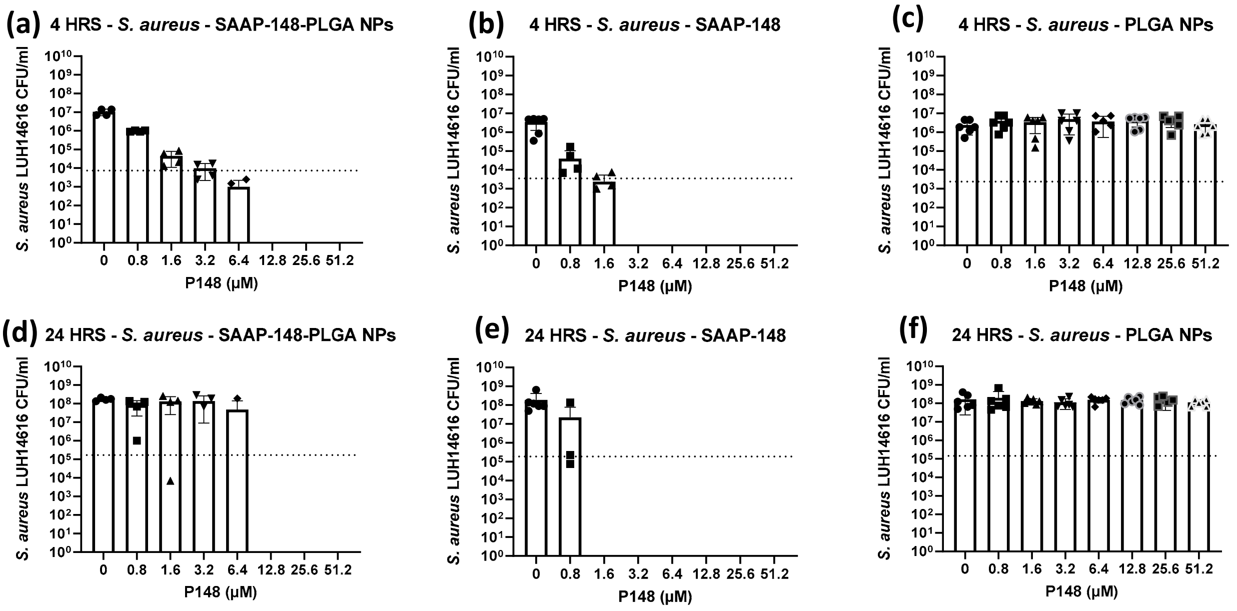

Figure S2. *In vitro* killing activities of SAAP-148-PLGA NPs (**a&d**), SAAP-148 (**b&e**), and PLGA NPs (**c&f**) against *A. baumannii (RUH875)* after 4 (**a-c**) and 24 hours (**d-f**) of incubation in 50% plasma at 37°C. D Dotted line represents the lethal concentration to kill 99.9% of bacterial counts ($LC_{99.9}$).

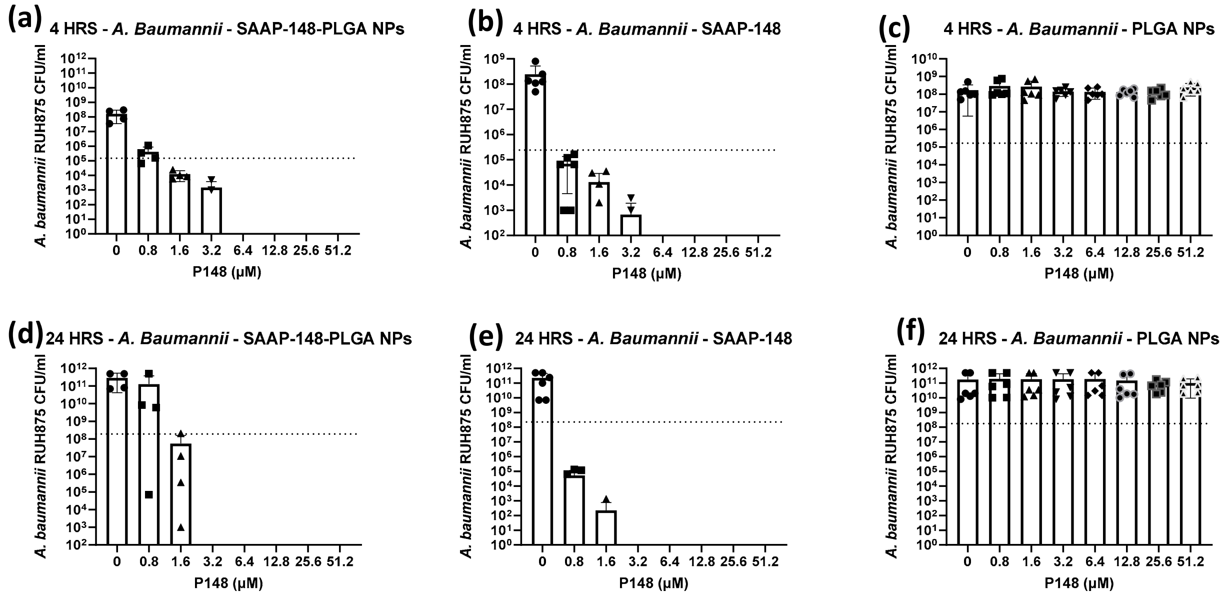

Table S1. *In vitro* cytotoxicity and hemolytic activity of SAAP-148 NPs. Cytotoxic and hemolytic activities upon exposure to SAAP-148 NPs, SAAP-148 solution, and blank NPs. Cytotoxicity against human skin fibroblasts is based on lacate dehydrogenase (LDH) assay and metabolic activity is based on water-soluble tetrazolium salt (WST-1) assay which were both performed in 0.5% human serum medium after 4 and 24 hrs incubation at 37°C with 95% humidity and 5% CO_2. Haemolytic activity is based on 5% hemolysis of human erythrocytes in PBS after 1 hr incubation at 37°C with 95% humidity and 5% CO_2. Data is retrieved from the median of three biological replicates, each performed in triplcates. Inhibitory concentration (IC_{50}) indicates the inhibitory concentration to induce 50% cytotoxicity.

	**LDH release IC_{50} (µM)**		**Metabolic activity IC_{50} (µM)**		**Hemolytic activity 5% (µM)**
**Cell type**	Human skin fibroblasts				Human eryhtrocytes
**Time (hrs)**	**4**	**24**	**4**	**24**	**1**
**SAAP-148 NPs**	>102.4	>102.4	>102.4	>102.4	8
**SAAP-148**	2.5	4.2	2.75	0.8	≤0.8
**Blank NPs**	>102.4	>102.4	>102.4	>102.4	15

Figure S3. Cytotoxicity upon exposure of 3D human skin equivalents to increasing concentrations halicin for 24 hrs based on lactate dehydrogenase (LDH) release of the subnatant from the 3D human skin equivalents. Cytotoxicity of halicin was both performed in PBS at 37°C with 95% humidity and 5% CO2. Data is retrieved from the mean of 1 experiment performed duplicate (n=1). Inhibitory concentration $(IC)_{50}$ indicates the inhibitory concentration to induce 50% cytotoxicity.

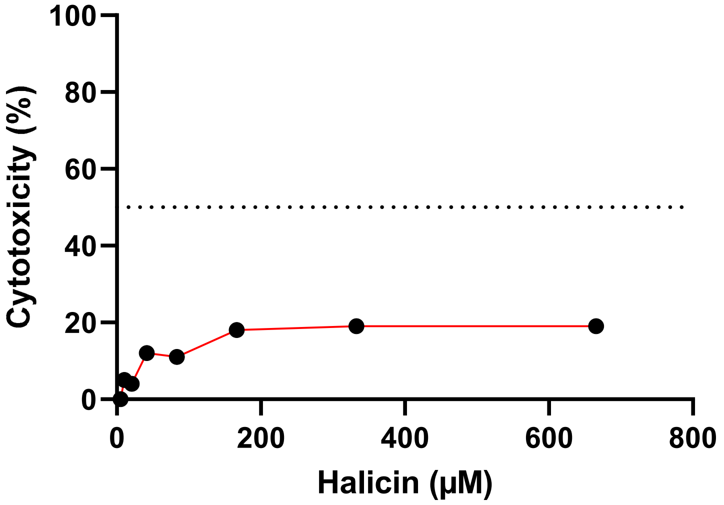

Supplement: Supplementary file 1 [file ijms-24-02867-s001.zip › ijms-2163916-SI.pdf]
